# Trichoepithelioma: A Rare but Crucial Dermatologic Issue

**Published:** 2014-07

**Authors:** Ali Akbar Mohammadi, Seyed Morteza Seyed Jafari

**Affiliations:** Shiraz Burn Research Center, Division of Plastic and Reconstructive Surgery, Department of Surgery, Shiraz University of Medical Sciences, Shiraz, Iran

**Keywords:** Trichoepithelioma, Diagnosis, Treatment

## Abstract

Trichoepithelioma is a rare benign skin lesion that originates from hair follicles. Trichoepitheliomas are mostly seen in the scalp, nose, forehead, and upper lip. We present a large family of Iranian origin with 15 individuals affected with multiple familial trichoepithelioma in four generations, and treated with three different methods. Trichoepithelioma is histologically similar to basal cell carcinoma and has a rare risk of malignant transformation. In addition, most frequent incidence of this disease in young to elderly women may lead to social and psychological issues. Precise diagnosis and management of this rare disease seem necessary.

## INTRODUCTION

Trichoepithelioma is a rare benign skin lesion that originates from hair follicles. Trichoepitheliomas are mostly seen in the scalp, nose, forehead, and upper lip. These skin lesions originate from benign proliferation of epithelial-mesenchymal origin cells.^1^^-^^6^ Trichoepitheliomas may be divided into the multiple familial trichoepithelioma, Solitary trichoepithelioma and desmoplastic trichoepithelioma.^7^ It usually appears as multiple lesions in the autosomal dominant type,^1^^,^^4^ or as an individual flesh colored papule or nodule measuring 2 to 8 mm in the sporadic type.^1^^,^^8^ Due to its autosomal dominant fashion, both genders receive the gene equally, but because of lessened expressivity and penetrance in men, more commonly seen in females.^5^ The gene for the development of familial trichoepithelioma encodes a tumor suppressor and links to the short arm of chromosome 9.^9^ In 1996, Harada *et al. *reported a mutation in this tumor suppressor encoding gene located on band 9q21 in multiple familial trichoepithelioma.^10^

Histopathologically, trichoepitheliomas contain branching nests of basaloid cells, horn cysts, and abortive hair papillae. The tumors represent benign hamartomas of the pilosebaceous apparatus.^11^ In some multiple familial trichoepitheliomas structures similar to basal cell carcinoma components (BCC) may be found.^11^^,^^12^

However, low incidence and lack of enough evidences, made trichoepithelioma management a controversial domain in dermatologic surgery. We present a large family of Iranian origin with 15 individuals affected with multiple familial trichoepithelioma in four generations, and treated with three different methods. All of these patients showed the typical clinical and histopathological features of multiple familial trichoepithelioma especially in face.

## CASE REPORT

**Fig. 1:**
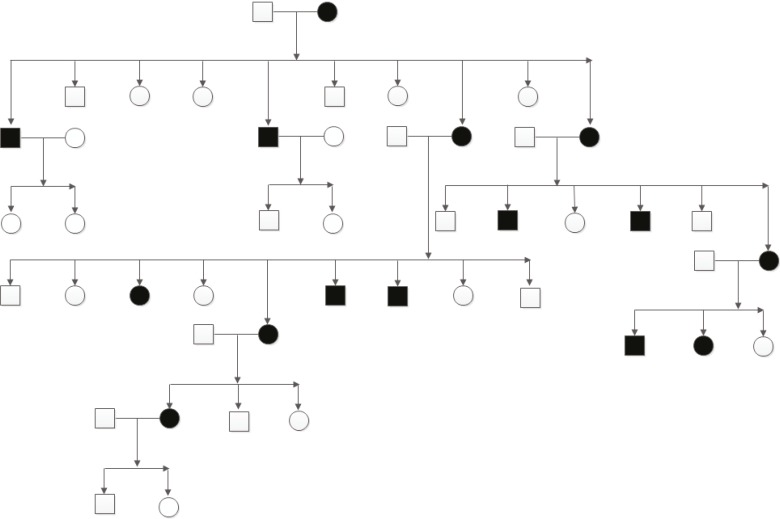
Family tree–15 members in this family affected with trichoepithelioma were shown in black

The family history showed that 15 individuals in four generations were similarly affected by trichoepithelioma (Figure 1).

**Fig. 2:**
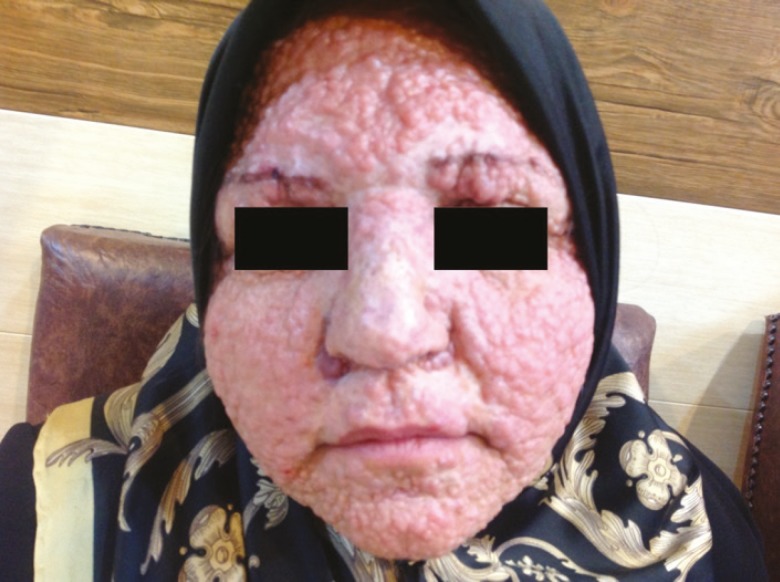
A 48-year old woman with trichoepithelioma of the upper lip, nasal dorsum and eyebrow areas that were electrocauterized 5 years ago with relatively acceptable results

From this family, 2 members were presented due to multiple facial tumors. The 48-year old woman stated that her facial lesions tended to become obvious after puberty. Her physical examination showed multiple firm skin-colored papules and nodules covering her face completely. In addition, a few papules or nodules were likewise found on her scalp. Her upper lip, nasal dorsum and eyebrow areas were electrocauterized 5 years ago with relatively acceptable results and she was seeking staged cauterization up to now (Figure 2). 

**Fig. 3:**
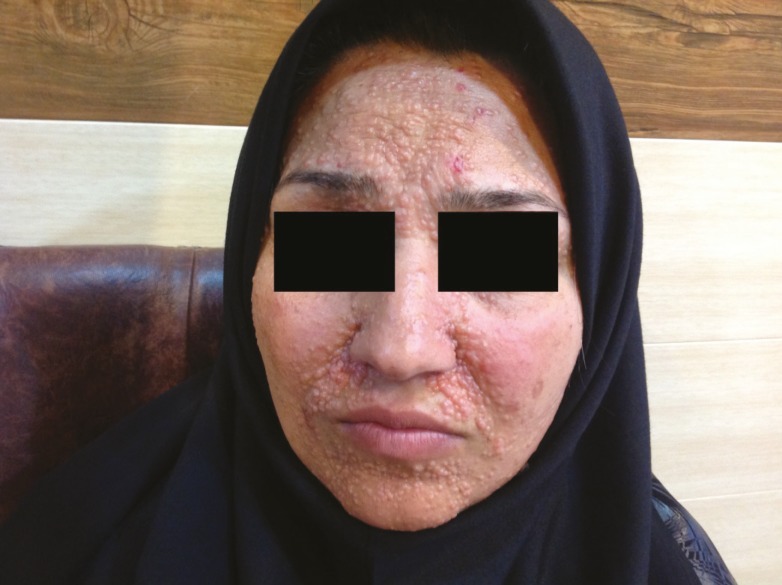
A 26-year old woman with trichoepithelioma

The 26-year old patient reported that her lesions appeared around her puberty. During her physical examination, we found multiple firm skin-colored papules and nodules covering the nasal area, the medial part of the eyebrows, and the peri-oral area. She did not have any scalp involvements; staged laser ablation was planned for her (Figure 3). Another patient in this family was treated with excision and unit reconstruction with skin grafting and somewhat satisfied.

## DISCUSSION

From one point of view, trichoepithelioma is significant because it histologically is similar to basal cell carcinoma, and although benign, there is a rare risk of malignant transformation.^1^^,^^4^^,^^13^ With CD34 positivity of the stromal cells around the nest favoring trichoepithelioma, immunohistochemical staining can sometimes be helpful in differentiating between trichoepithelioma and basal cell carcinoma.^1^ Moreover, trichoepithelioma mostly present papillary-mesenchymal bodies, granulomas, and calcification, while basal cell carcinoma is more likely to present inflammation, retraction, a higher frequency of apoptotic cells, and mitotic figures.^1^^,^^8^

On the other hand, although both genders receive the gene equally, most patients are young to aging adult women due to lessened expressivity and penetrance in men. Furthermore, these lesions are annoying and might cause social and psychological problems because of the facial involvement. Moreover, selection of inappropriate methods may cause incomplete treatment and disease recurrence and excessive treatment may result in increased risk of cosmetic and functional compromise.^3^

As a result, to avoid over-treatment and unnecessary complications, selection of proper choice of treatment according to the depth of the lesions is crucial.^3^ In our study, three patients were treated by three different methods (electrodessication, skin transplantation, and laser therapy). Although different methods have been proposed for trichoepithelioma management, low incidence and lack of enough evidences made it a controversial domain in dermatologic surgery. All in all, as a benign tumor, trichoepithelioma can be managed safely with surgical removal. Alternatives include dermal abrasion and laser surgery, curettage although these options may be associated with an increased rate of recurrence.^7^^,^^14^ Furthermore, a Brazilian study of several types of cutaneous tumors described the use of topical 5% imiquimod cream in trichoepitheliomas for 32 weeks. Trichoepitheliomas were treated with imiquimod and retinoic acid presented 80% clearance.^15^ It should be mentioned that consideration of depth of the skin lesions is important in management of such lesions, and some patients might require skin grafting after excision of lesions.

Trichoepithelioma is histologically is similar to basal cell carcinoma, and has a rare risk of malignant transformation. In addition, most frequent incidence of this disease in young to elderly women may lead to social and psychological issues. Therefore, precise diagnosis and early management of this rare disease with different approved methods is necessary. In addition, a multicenter clinical study on trichoepithelioma patients is demanded to compare efficacy and failure of different treatment methods.

## CONFLICT OF INTEREST

The authors declare no conflict of interest. 

## Footenote

Please cite this paper as:

Mohammadi AA, Seyed Jafari SM. Trichoepithelioma: A Rare but Crucial Dermatologic Issue. World J Plast Surg 2014;3(2):142-145.
